# Mitigating Nitrous Oxide Emissions from Tea Field Soil Using Bioaugmentation with a *Trichoderma viride* Biofertilizer

**DOI:** 10.1155/2014/793752

**Published:** 2014-04-06

**Authors:** Shengjun Xu, Xiaoqing Fu, Shuanglong Ma, Zhihui Bai, Runlin Xiao, Yong Li, Guoqiang Zhuang

**Affiliations:** ^1^Research Center for Eco-Environmental Sciences, Chinese Academy of Sciences, Beijing 100085, China; ^2^Institute of Subtropical Agriculture, Chinese Academy of Sciences, Changsha 410125, China

## Abstract

Land-use conversion from woodlands to tea fields in subtropical areas of central China leads to increased nitrous oxide (N_2_O) emissions, partly due to increased nitrogen fertilizer use. A field investigation of N_2_O using a static closed chamber-gas chromatography revealed that the average N_2_O fluxes in tea fields with 225 kg N ha^−1^ yr^−1^ fertilizer application were 9.4 ± 6.2 times higher than those of woodlands. Accordingly, it is urgent to develop practices for mitigating N_2_O emissions from tea fields. By liquid-state fermentation of sweet potato starch wastewater and solid-state fermentation of paddy straw with application of *Trichoderma viride*, we provided the tea plantation with biofertilizer containing 2.4 t C ha^−1^ and 58.7 kg N ha^−1^. Compared to use of synthetic N fertilizer, use of biofertilizer at 225 kg N ha^−1^ yr^−1^ significantly reduced N_2_O emissions by 33.3%–71.8% and increased the tea yield by 16.2%–62.2%. Therefore, the process of bioconversion/bioaugmentation tested in this study was found to be a cost-effective and feasible approach to reducing N_2_O emissions and can be considered the best management practice for tea fields.

## 1. Introduction

Three typical land-uses occur in most areas of southern subtropical China: woodlands in the mountains, uplands on slopes, and paddy fields in the lowlands [1]. In China, 1.3 M ha of tea plantations has been established during the last 50 yrs., due to rapid economic development [2]. Most of these recent plantations occupy large areas that were originally woodlands and are continuing to expand [3]. Land-use conversion is typically characterized by frequent tillage and use of large amounts of nitrogenous fertilizer, which contributes significantly to losses of N as nitrous oxide (N_2_O) emissions to the atmosphere [4]. N_2_O emissions are, on average, >10 times higher from tea fields than from forests. N_2_O has an ozone-depleting potential similar to that of hydrochlorofluorocarbons and a global warming potential 300 times that of CO_2_ [3, 5, 6].

In China, annual production of rice straw was about 1.74 × 10^8^ t in 2000, containing about 2.5 − 4.0 × 10^4^ t N [7]. Due to lack of feasible conversion technologies, until the 2000s, on-site burning was traditionally used to dispose of crop residues, resulting in severe environmental pollution including greenhouse gases and nitrogen oxides [8]. Recently, straw return has become the preferred method of rice straw disposal because of its positive effects on crop yield and soil properties, such as increased soil organic carbon content. However, straw decomposition in soils takes time; over the short-term decomposition, straw incorporation can hamper crop root penetration, causing N deficiencies and contributing to disease and weed problems [9, 10], directly limiting its application in agriculture. Therefore, it is important to develop a method for enhancing straw decomposition before or after incorporation.

Many studies have attempted to hasten straw decomposition via microbial processes.* Trichoderma* spp. is the best-known cellulolytic fungi that can accelerate the process of rice straw decomposition and produce more soil-available nutrients such as labile organic matter and inorganic N/P/S [11–13]. However, a major challenge in implementing bioconversion of rice straw is identifying a suitable carrier substrate to amplify the target organism, promote survival of* Trichoderma* spp. in the rice straw, and enhance colonization in the soil near plant roots. Fortunately, sweet potato starch wastewater (SPSW) from local starch production plants could be used as an appropriate culture medium for microbial growth because of its high organic content. A few studies have been published on production of microbial biomass through starch industry wastewater with various microbes such as* Trichoderma viride* [14].

Compared to chemical fertilizers and protectants, some strains of* Trichoderma* spp. provide additional benefits in terms of plant growth and productivity, such as systemic resistance to disease and abiotic stresses such as water deficits and salt stress [15, 16]. If* Trichoderma* spp. colonize the roots, they strongly affect plant physiology by changing plant gene expression, providing season-long benefits to the plants [17, 18]. Another major benefit is induction of increased N use efficiency (NUE) in plants [19], potentially reducing the N fertilizer application rate by 30%–50% through bioconversion/bioaugmentation with no reduction in yield. Therefore, the lower N inputs to soil and higher NUE may reduce N_2_O emissions.

The objectives of this study were to (1) develop a cost-effective technology for using SPSW and rice straw for large-scale production of* Trichoderma viride* biofertilizer, (2) evaluate the feasibility of biofortification of crops through application of* T. viride* N fertilizers to reduce N_2_O emissions, and (3) minimize synthetic N fertilizer input via recycling of N to achieve a sustainable agricultural system.

## 2. Methods and Materials

### 2.1. Site Description

**Figure 1:**
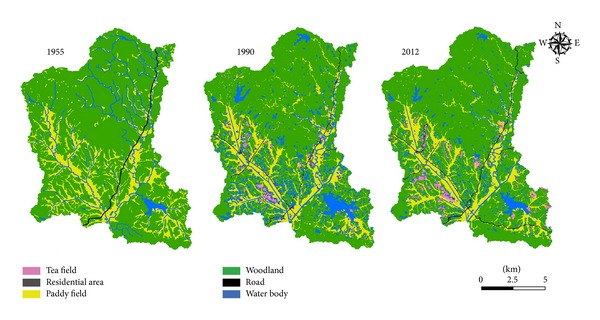
Land-use distributions during 1955–2012.

The field experiment was carried out at the Changsha Research Station for Agricultural and Environmental Monitoring (CRSAEM), Changsha, Hunan, in subtropical central China (28°32′50′′N, 113°10′58′′E). The region has a subtropical monsoon climate with a mean annual air temperature of 17.5°C and a mean annual precipitation of 1330 mm (1979–2010). The red soils of this area are classified as ultisols (USDA soil taxonomy) [20]. The selected site is a typical hilly, agricultural catchment with pine forests (woodlands), paddy fields (lowland), and tea fields (upland) as the three primary land-use types, accounting for 65.5, 25.1, and 3.4% of the total catchment area (135 km^2^, Figure 1), respectively. The other small upland areas in the catchment are cropped sweet potato (<0.5%). The land-use distribution in 1990 shows substantial conversion from woodlands dominated by Masson pine and bamboo to large-scale tea plantations (*Camellia sinensis* L., Figure 1). Each land-use type is fertilized other than the woodlands. The average annual application rate of N fertilizers was 450, 300, and 250 kg N ha^−1^ yr^−1^ for tea fields, paddy fields, and sweet potato fields, respectively.

### 2.2. Preparation of Biofertilizer

#### 2.2.1. Raw Materials and Starter Inoculum

SPSW, containing approximately 25 g L^−1^ COD and 1 g L^−1^ total N, was obtained from a local sweet potato starch production company (Xiangfeng Corp., Hunan, China) as a by-product of sweet potato starch processing. Rice straw (total organic carbon (TOC), 56.5%; total nitrogen (TN), 0.46%) was collected after harvest from local paddy fields and ground to 5–8 mm lengths for the solid fermentation culture of* Trichoderma viride*.

The strain* T. viride *EBL13 was isolated from the soil and was found to be active against phytopathogenic fungi in our laboratory [21]. The starter cultures were obtained by inoculating 500 *μ*L of a spore suspension at 10^8^ spores mL^−1^ into 20 mL potato dextrose broth (PDB; Sigma-Aldrich, St. Louis, MO) and incubating at 25°C in orbital agitation at 150 rpm in the dark for 48 h.

#### 2.2.2. Mass Production of* T. viride* Mycelium

The starter cultures (2 mL) were transferred to a 500-mL Erlenmeyer flask containing 150 mL sterile sweet starch industry wastewater (121 ± 1°C for 15 min) and were incubated in a rotary shaker at 28 ± 1°C and 200 ± 5 rpm for 48 h. The mycelium was collected by centrifugation at 4000 g for 10 min, the biomass fresh weight was determined, and then the fungal biomass was prepared for solid-state fermentation (SSF) with rice straw.

#### 2.2.3. Production of Biofertilizer by Solid-State Fermentation

The homogenized mycelial mat (10 g dewatered mycelium with 10 mL fermentation supernatant) was transferred to a 500-mL Erlenmeyer flask containing 10 g sterile rice straw (121 ± 1°C for 15 min). Afterwards, the flasks were incubated at 28 ± 1°C for 148 h until mass production of* T. viride *conidia occurred. The conidia concentration was measured as cfu g^−1^ air-dried cotton stalk substrate, using a modified conidia assessment method [22].

### 2.3. Biofertilizer Application to the Tea Plantation

Biofertilizer was compared with chemical fertilizer (N) using a completely randomized design in an ongoing trial of about 3-year-old tea (*Camellia sinensis cv.*) from November 2010 to May 2012 at a tea plantation in Jinjing. There were four treatments in the field experiment: (1) unfertilized (CK0), (2) chemical fertilizer (CNH450, urea applied at 450 kg N ha^−1^ yr^−1^; CNL225, fertilizer applied at 225 kg N ha^−1^ yr^−1^), (3) biofertilizer (BFH225, biofertilizer applied at 50000 kg ha^−1^ yr^−1^, with the total N adjusted to that of CNL225; BFL113, biofertilizer applied at 25000 kg ha^−1^ yr^−1^, with the total N adjusted to half that of CNL225), and (4) raw materials (RMH225, sweet starch industry wastewater and rice straw at 50000 kg ha^−1^ yr^−1^ for fermentation of biofertilizer; RML113, sweet starch industry wastewater and rice straw at 25000 kg ha^−1^ yr^−1^). The fertilizer was applied in three stages each year: 20% of the total N on November 12, 60% on March 1, and 20% on October 1, tilled 10–15 cm under the soil surface. The experimental plot was 60 m^2^ (10 × 6 m^2^) for each fertilizer treatment.

### 2.4. Field Measurement of N_2_O Fluxes

Soil N_2_O fluxes were measured using a static closed chamber and gas chromatography (GC), as described by Li et al. [3] and Zheng et al. [23], from March 2011 to April 2012. The closed mini-chambers were constructed of polyvinylchloride, 0.15 m in diameter and 0.18 m in length, with sharpened ends and screw lids fitted with rubber septa for gas sampling. The chambers were gently inserted vertically into the soil to a depth of 0.05 m using the sharpened ends. Headspace gas samples were collected from 09:30 to 10:30 a.m. once a week during tea harvest seasons (spring tea: mid-March to mid-April; autumn tea: mid-September to mid-October). For each treatment, 3 replicate gas samples were collected from the headspace into preevacuated 12-mL vials (Exetainers, Labco, High Wycombe, UK) 0 and 30 min after the lid was closed. After gas sampling, the air temperature in each chamber was measured for subsequent correction of the flux calculation. The N_2_O concentrations in the gas samples were analyzed using a gas chromatograph (Agilent 7890A, Agilent, Santa Clara, CA) fitted with a ^63^Ni-electron capture detector and an automatic sample injector system [3]. N_2_O fluxes (FLUX30, g N ha^−1^ d^−1^) were calculated using the following equation:

(1) FLUX30=(c30−c0)·MN2OV0·hΔt·T0T0+Tair·100001000·24·2·MNMN2O,

where *c*_30_ − *c*_0_ is the difference in the N_2_O concentration in the headspace of the mini-chamber 0 and 30 min after the lid was closed (ppmv); *M*_N_2_O_ is the molecular weight of N_2_O (g mol^−1^); *V*_0_ is the molecular volume of N_2_O under standard conditions (temperature = 273 K and pressure = 1013 hPa), 22.4 × 10^−3^ m^3^; *T*_0_ = 273 K; *M*_N_ is the atomic weight of nitrogen (g mol^−1^); *h* is the chamber height (m); Δ*t* is the incubation period (0.5 h); *T*_air_ is the air temperature inside the mini-chamber (°C); and 10,000, 1000, and 24 are conversion factors for m^2^ to ha, mg to g, and h to days, respectively.

### 2.5. Auxiliary Field Measurements

In addition to measuring gas fluxes, ammonium-N and nitrate-N in the soil were measured along with tea plant growth. Four 0–20 cm soil samples were collected from random locations in each plot using a soil auger during each field measurement of N_2_O fluxes. Each fresh soil sample was manually homogenized and analyzed for soil ammonium-N (NH_4_^+^-N), nitrate-N (NO_3_^−^-N), and total N using an automated flow injection analyzer (FIAStar 5000, Foss Tecator, Hoganas, Sweden) as described by Liu et al. [24]. Tea plant growth was monitored by recording the weight of a fresh tea bush (bud with two leaves) during the tea harvest seasons (spring tea: mid-March to mid-April; autumn tea: mid-September to mid-October).

### 2.6. Statistical Analyses

All statistical analyses were conducted using SPSS 12.0 (SPSS China, Beijing, China) and Origin 8.0 (Origin Lab Ltd., Guangzhou, China). The statistical significance of the results was determined using Duncan's multiple-range test (*P* < 0.05). Simple correlation coefficients between soil N_2_O flux and NH_4_^+^-N and NO_3_^−^-N contents were calculated using the same statistical package.

## 3. Results and Discussion

### 3.1. Effects of Land-Use Changes on N_2_O Emissions from Hilly Areas of Subtropical Central China

Figure 1 depicts changes in land-use distribution patterns using a geographic information system since 1955. In the late 1950s, the land-use pattern was simple and mainly comprised of woodlands and paddy fields with 63.05 and 29.38% of the total land, respectively. With rapid economic development and population increases, rapid land-use and land-cover changes have taken place in these areas beginning in the 1990s; there has been a large decline in forest cover due to tea plantation expansion. Overall, 398 ha of woodland areas has been converted to tea crop cultivation, about 2.98% of the total area of the district. Woodland cover (60.3%) has decreased by 4.30% compared to 1955. By 2012, tea plantation land had expanded to 3.42% of the total area of the district, a marked increase of 15.2% from 1990 to 2012.

Under the typical fertilization model for tea plantations, N_2_O fluxes associated with high-N treatment (450 kg N ha^−1^ yr^−1^) in the spring and autumn of 2011 and the spring of 2012 were 24.8–49.0, 15.2–40.2, and 28.6–37.6 g N ha−1 d−1, respectively. For low-N fertilization (225 kg N ha^−1^ yr^−1^), N_2_O fluxes during the same periods were 6.77–18.4, 13.5–28.2, and 10.7–15.2 g N ha^−1^ d^−1^, respectively. However, for woodlands in the same district, N_2_O fluxes during the same periods were −8.21–11.1, 0.85–2.12, and 1.99–2.39 g N ha^−1^ d^−1^, respectively. Therefore, the N_2_O fluxes from tea plantations with high-N (450 kg N ha^−1^ yr^−1^) and low-N (225 kg N ha^−1^ yr^−1^) application were 17.7 ± 3.4 and 9.4 ± 6.2 times higher than those from woodlands, respectively. Many studies have reported that land-use changes can impact N_2_O emissions and that N_2_O emissions significantly increase when woodlands are converted into pastures [25], orchards [26], or cropland [27]. Merino et al. [28] measured N_2_O releases from cropland and a pasture (7.40 and 13.15 g N ha^−1^ d^−1^) and found that they were 3 and 6 times higher than those from a forest (2.19 g N ha^−1^ d^−1^), respectively. We have previously reported the high annual variability of N_2_O emissions as well as its spatial variability and distribution from a tea field in 2010 [3, 29]. Many studies have shown that agricultural land has much higher N_2_O emissions than forests because of generally higher N in soils that may be further enhanced through intensification of the N cycle through application of synthetic N fertilizer [30, 31].

### 3.2. Bioconversion of Sweet Potato Starch Wastewater and Rice Straw into Biofertilizer by* Trichoderma viride*

In the studied catchment, paddy fields accounting for 25.1% of the total land area produced 3.02 × 10^4^ t yr^−1^ straw containing 9.07 × 10^3^ t C and 1.21 × 10^2^ t N (Table 1). Sweet potatoes were cultivated in the upland area, making up 0.25% of the total land area of the catchment. The sweet potatoes were processed to produce 150 t yr^−1^ refined starch, during which about 1.2 × 10^4^ t SPSW was produced containing 252 t C and 9.6 t N (Table 1). The SPSW was found to be suitable for cultivation of* T. viride*, with a dry mycelium weight of about 8.96 g L^−1^. About 73.6% of the C and 81.3% of the N in the wastewater were transferred into the mycelium. To optimize conditions and enhance straw decomposition, the mass* T. viride* mycelium was mixed with rice straw by solid-state fermentation to produce biofertilizer with a maximum conidia concentration of 3.2 × 10^10^ cfu g^−1^. By liquid-state fermentation of the SPSW and solid-state fermentation of the paddy straw, the tea plantation can be provided with 1.1 × 10^3^ t C yr^−1^ and 27.0 t N yr^−1^ or 2.4 t C ha^−1^ yr^−1^and 58.7 kg N ha^−1^ yr^−1^ over the 460 ha catchment area (calculated from Table 1). Harman reported that* Trichoderma* strains can stabilize soil nutrients, enhance nutrient uptake, promote root development, increase root hair formation, and induce systemic resistance to biotic stresses (diseases) and abiotic stresses (water deficits) [15, 16, 18, 21]. The biological activity of* Trichoderma* spp. could replace some functions of synthetic nitrogen to minimize synthetic inputs of fertilizer in tea cultivation.

### 3.3. Mitigation Options for Reducing N_2_O Emissions

**Figure 2:**
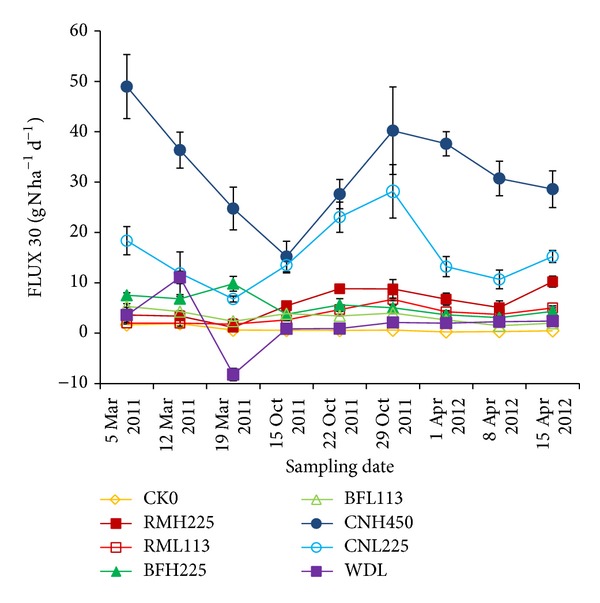
Variations in soil N_2_O flux in tea fields during 2011-2012 (CK0: unfertilized; RMH225: raw materials applied at 225 kg N ha^−1^ yr^−1^; RMH113: raw materials applied at 113 kg N ha^−1^ yr^−1^; BFH225: biofertilizer applied at 225 kg N ha^−1^ yr^−1^; BFH113: biofertilizer applied at 113 kg N ha^−1^ yr^−1^; CNH450: urea applied at 450 kg N ha^−1^ yr^−1^; and CNH225: urea applied at 225 kg N ha^−1^ yr^−1^, WDL-woodland).

Table 2 and Figure 2 show that application of synthetic N fertilizer significantly increased tea yields and also N_2_O emissions compared to unfertilized fields. Addition of synthetic N fertilizer as CNL225 and CNL450 increased the average tea yields by 9.1% and 39.6% (spring tea, 2011), 39.9% and 111.8% (autumn tea, 2011), and 64.2% and 103.3% (spring tea, 2012), respectively, compared to the unfertilized treatment (CK0). At the same time, fluxes of N_2_O were significantly affected by the rate of synthetic N fertilization. Based on measurements in 2011-2012, the fertilization treatments CNL225 and CNL450 significantly stimulated N_2_O emissions by 849% and 2881% (spring tea, 2011), 3686% and 4731% (autumn tea, 2011), and 3889% and 10250% (spring tea, 2012), respectively. These results are consistent with those of previous studies indicating that nitrogen fertilizer application enhances emissions of N_2_O from agricultural fields [26, 32–34]. In addition, the magnitude of the N_2_O emissions from our tea fields was much higher than previously reported values for paddy fields and uplands with N fertilization. Accordingly, it is urgent to establish technologies and management practices for mitigating N_2_O emissions from tea fields while sustaining or increasing tea production.

Improved management using biofertilizer instead of synthetic N fertilizer significantly decreased N_2_O emissions (Figure 2). The BFH225 treatment reduced N_2_O emissions by 33.3%–71.8% and increased the tea yield by 16.2%–62.2% compared to the CNL225 treatment, depending on the season. The RMH225 treatment also reduced N_2_O emissions by 43.6%–80.0%; however, the yield of 2012 spring tea decreased and some tea plants died. There have been contradictory reports on the effects of organic waste materials on N_2_O emissions. Some studies have shown an inhibitory effect [35–37], while others have reported stimulation [38, 39]. Based on our results, direct application of the organic waste materials (rice straw and starch industry wastewater) to the tea field carried significant risks to the tea plants. However, transferring these waste materials into biofertilizer by fermentation was a feasible approach for decreasing N_2_O fluxes from tea fields.

In addition, compared to the high application rate of chemical N fertilizer (CNL450), the BFH225 treatment that reduced the annual N fertilization rate by 50% did not affect the tea yield (Table 2) but significantly decreased average N_2_O emissions by 71.6% (Figure 2). This decrease may be ascribed to three possible mechanisms. First, because* Trichoderma viride* could promote plant growth, mineral nitrogen in the soil may be more quickly taken up by the vigorously growing tea plants in the presence of* Trichoderma *spp. [19], thereby reducing the N in the substrate available for N_2_O production. Second, application of biofertilizer with a high C/N ratio (>30) results in temporarily improving soil N immobilization and thus a low availability of soil N for N_2_O emission [40]. Finally, the buffering and loosening effect of the organic matter in the biofertilizer resulting in higher pH and oxygen in the soils may have favored N_2_ as an end product rather than N_2_O in denitrification [41].

### 3.4. Effects of Soil NH_4_^+^ and NO_3_^−^ Concentrations on N_2_O Production

**Figure 3:**
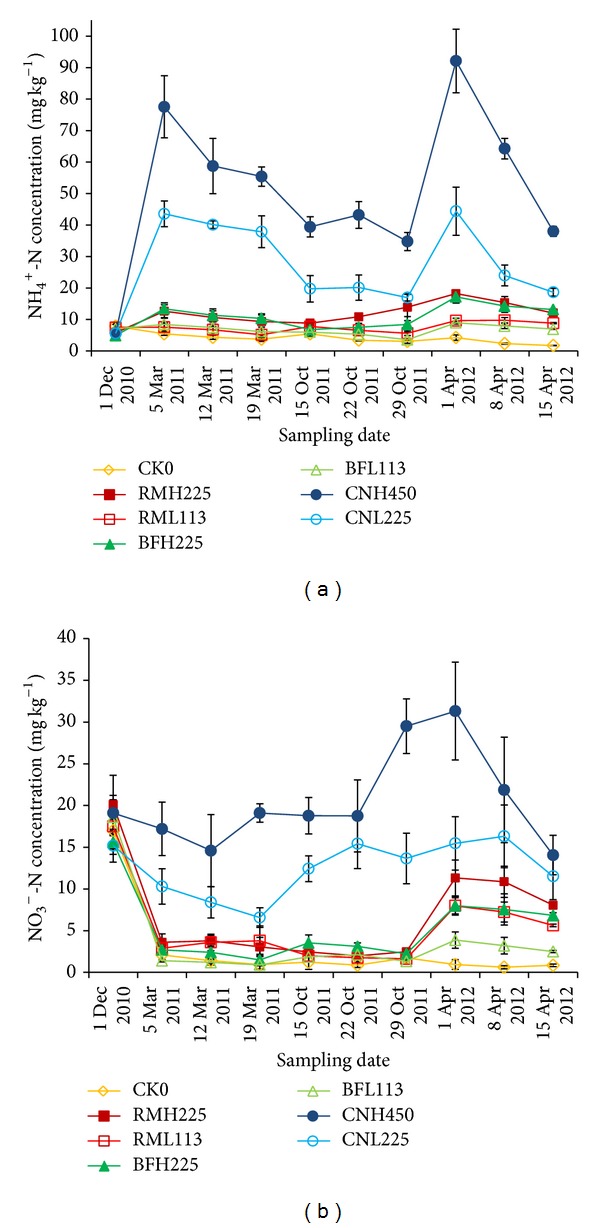
Variations in soil concentrations of (a) NH_4_^+^-N and (b) NO_3_^−^-N in tea fields during 2010-2012 (CK0: unfertilized; RMH225: raw materials applied at 225 kg N ha^−1^ yr^−1^; RMH113: raw materials applied at 113 kg N ha^−1^ yr^−1^; BFH225: biofertilizer applied at 225 kg N ha^−1^ yr^−1^; BFH113: biofertilizer applied at 113 kg N ha^−1^ yr^−1^; CNH450: urea applied at 450 kg N ha^−1^ yr^−1^; and CNH225: urea applied at 225 kg N ha^−1^ yr^−1^).

N fertilizer application significantly increased the soil (0–20 cm) NH_4_^+^ and NO_3_^−^ concentrations over those of unfertilized soil (Figure 3). For CNL450 and CNL225, the NH_4_^+^ concentrations varied between 17.0 and 92.1 mg N kg^−1^ soil (dw), with mean concentrations as high as 56.1 ± 19.6 and 29.5 ± 11.9 mg N kg^−1^, respectively. The NO_3_^−^ concentrations ranged from 6.6 to 31.3 mg N kg^−1^ and averaged 20.6 ± 6.1 and 12.2 ± 3.4 mg N kg^−1^ for CNL450 and CNL225, respectively. For BFH225 and BFH113, the NH_4_^+^ concentration ranged from 3.6 to 17.2 mg N kg^−1^ with lower mean concentrations of 11.4 ± 3.4 and 6.8 ± 1.7 mg N kg^−1^ throughout the tea season. NO_3_^−^ concentrations varied between 0.9 and 8.0 mg N kg^−1^ with mean concentrations of 4.2 ± 2.5 and 2.1 ± 1.0 mg N kg^−1^ . N concentrations in the CNL450 and CNL225 treatments decreased rapidly after fertilization; however, in the BFH225 and BFH113 treatments, it remained comparatively stable. In addition, the N concentrations in the BFH113 treatment were lower than in the BFH225 treatment with more intensive fertilization. Organic nitrogen slowly released from the biofertilizer may have been responsible for this phenomenon.

**Figure 4:**
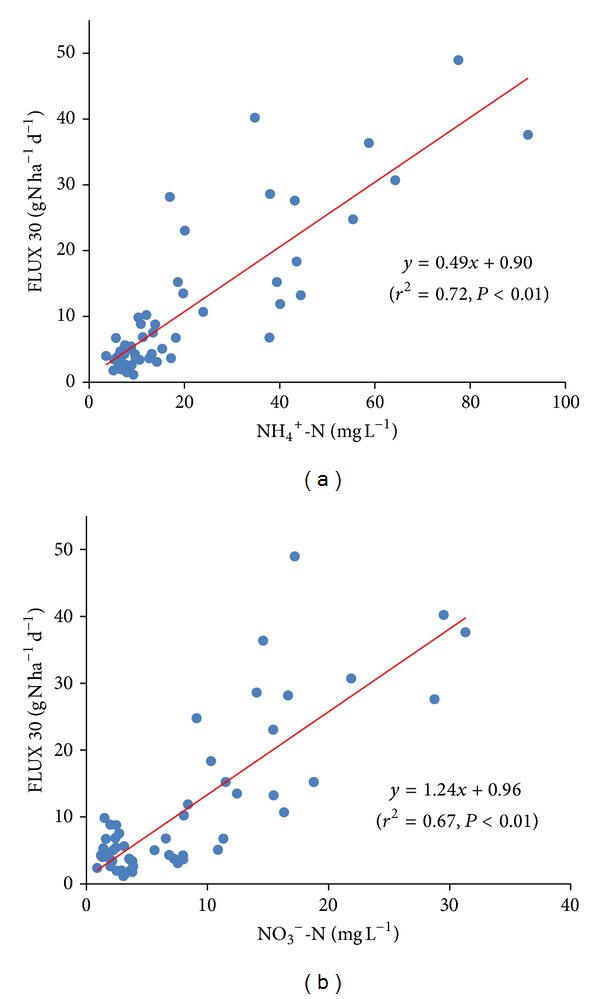
Relationships between N_2_O emissions from the tea field and soil concentrations of (a) NH_4_^+^-N and (b) NO_3_^−^-N.

A significant positive relationship was found when N_2_O emissions were linearly regressed against soil N concentrations across the various treatments. For all N fertilizer treatments, a significant positive correlation existed between N_2_O fluxes and soil NH_4_^+^-N contents (*y* = 0.49*x* + 0.90, *r* ^2^ = 0.72, and *P* < 0.01, Figure 4(a)) and a similar effect was observed between N_2_O fluxes and soil NO_3_^−^-N contents (*y* = 1.24*x* + 0.96, *r* ^2^ = 0.67, and *P* < 0.01, Figure 4(b)).

In several previous studies, soil temperature, soil moisture, and NH_4_^+^-N or NO_3_^−^-N contents were identified as the main environmental drivers of N_2_O fluxes [26, 34, 42], affecting either nitrification or denitrification and thus N_2_O production. The temperature and humidity of the soil, which were significantly correlated with N_2_O fluxes, are controlled by natural factors such as seasons and precipitation. However, another key factor affecting N_2_O flux, the N content in the soil, can be controlled by changing the type of fertilizer applied, the fertilization rate, and the timing of application, providing a feasible method of controlling the N_2_O flux.

## 4. Conclusions

Land-use conversion from woodlands to tea fields in subtropical areas of central China leads to increased N_2_O emissions, partly due to increased N fertilizer use. By liquid-state fermentation of SPSW and solid-state fermentation of paddy straw by* T. viride*, tea plantations can be provided with biofertilizer that can replace some functions of synthetic N, minimizing synthetic inputs of fertilizer in tea cultivation. Improved management using biofertilizer rather than synthetic N fertilizer can significantly decrease N_2_O emissions while sustaining or increasing tea production. Thus, the process described here using SPSW and rice straw for mass-scale production of* T. viride *biofertilizer is a feasible and cost-effective approach for minimizing synthetic inputs of fertilizer, reducing cumulative N_2_O emissions and developing the best management practices for N in soils.

## Figures and Tables

**Table 1:** Process parameters for bioconversion of SPSW and rice straw into biofertilizer by* T. viride*.

Parameters	Raw materials		*T. viride *	Biofertilizer
	Rice straw	SPSW	Mycelium	
Water content (%)	5.3	97.8	81	48.3
Total organic carbon (%)	56.5	2.1	14.4	18.4
Total nitrogen (%)	0.4	0.08	0.65	0.45
Dry mycelium weight (g L^−1^)	—	—	8.96	—
Conidia concentration (cfu g^−1^)	—	—	—	3.2 × 10^10^

Note: “—” means not test.

**Table 2:** Productivity of tea fields (fresh tea, kg ha^−1^) with various fertilizer treatments, 2011-2012.

Treatment	2011 spring tea (kg ha^−1^)			2011 autumn tea (kg ha^−1^)			2012 spring tea (kg ha^−1^)		
	March 5	March 12	March 19	October 15	October 22	October 29	April1	April 8	April 15
CK0	125.4 ± 15.6^a^	306.6 ± 11.2^a^	377.3 ± 35.4^a^	269.8 ± 15.9^a^	255.4 ± 65.4^a^	231.4 ± 45.3^a^	207.2 ± 10.9^a^	285.9 ± 49.9^a^	508.2 ± 54.4^a^
RMH225	169.6 ± 23.8^bc^	347.3 ± 51.9^ab^	545.8 ± 49.6^c^	413.4 ± 14.6^bc^	371.2 ± 24.8^bc^	313 ± 37.0^b^	289.5 ± 16.3^a^	301.7 ± 36.9^ab^	457.8 ± 36.3^a^
RML113	137.9 ± 21.3^ab^	239.2 ± 41.5^ab^	394.7 ± 40.3^a^	355.5 ± 28.3^b^	318.9 ± 28.3^b^	292.6 ± 19.6^ab^	255.9 ± 57.2^a^	268.7 ± 55.5^ab^	468.7 ± 13.7^a^
BFH225	202.9 ± 40.7^c^	426.3 ± 74.0^b^	784.5 ± 44.6^d^	620.3 ± 39.6^d^	587.4 ± 43.3^d^	463.5 ± 39.1^c^	400.4 ± 24.9^c^	528.4 ± 87.0^c^	783.9 ± 147.7^b^
BFL113	142.2 ± 12.5^ab^	292.7 ± 9.5^ab^	521.4 ± 31.2^bc^	453.9 ± 34.7^c^	387.5 ± 8.3^c^	327.1 ± 34.7^b^	282.3 ± 54.9^a^	364.6 ± 40.2^b^	573.5 ± 34.7^ab^
CNH450	202.0 ± 4.9^c^	349.3 ± 45.9^ab^	542.1 ± 16.8^c^	575.5 ± 62.5^d^	532.9 ± 30.5^d^	494 ± 46.0^c^	530.3 ± 37.9^c^	633.5 ± 27.7^*c*^	672.7 ± 121.3^b^
CNL225	145.6 ± 12.4^ab^	309.3 ± 56.9^ab^	416.6 ± 30.2^ab^	388.8 ± 67.0^bc^	350.3 ± 4.4^bc^	320.3 ± 36.9^b^	448 ± 34.3^b^	487.2 ± 22.8^b^	538.9 ± 45.3^ab^

Note: in each row, means followed by the same letter are not significantly different at *P* < 0.05.
